# A SSVEP Stimuli Encoding Method Using Trinary Frequency-Shift Keying Encoded SSVEP (TFSK-SSVEP)

**DOI:** 10.3389/fnhum.2017.00278

**Published:** 2017-06-02

**Authors:** Xing Zhao, Dechun Zhao, Xia Wang, Xiaorong Hou

**Affiliations:** ^1^Department of Biomedical Engineering, School of Bioinformatics, Chongqing University of Post and TelecommunicationsChongqing, China; ^2^Department of Health Information Management and Decision Making, College of Medical Informatics, Chongqing Medical UniversityChongqing, China

**Keywords:** SSVEP, BCI, EEG, HMI, FSK-SSVEP, TFSK-SSVEP

## Abstract

SSVEP is a kind of BCI technology with advantage of high information transfer rate. However, due to its nature, frequencies could be used as stimuli are scarce. To solve such problem, a stimuli encoding method which encodes SSVEP signal using Frequency Shift–Keying (FSK) method is developed. In this method, each stimulus is controlled by a FSK signal which contains three different frequencies that represent “Bit 0,” “Bit 1” and “Bit 2” respectively. Different to common BFSK in digital communication, “Bit 0” and “Bit 1” composited the unique identifier of stimuli in binary bit stream form, while “Bit 2” indicates the ending of a stimuli encoding. EEG signal is acquired on channel Oz, O1, O2, Pz, P3, and P4, using ADS1299 at the sample rate of 250 SPS. Before original EEG signal is quadrature demodulated, it is detrended and then band-pass filtered using FFT-based FIR filtering to remove interference. Valid peak of the processed signal is acquired by calculating its derivative and converted into bit stream using window method. Theoretically, this coding method could implement at least 2^*n*−1^ (*n* is the length of bit command) stimulus while keeping the ITR the same. This method is suitable to implement stimuli on a monitor and where the frequency and phase could be used to code stimuli is limited as well as implementing portable BCI devices which is not capable of performing complex calculations.

## Introduction

“Brain-Computer Interface” (BCI) is a very attractive technology which provides hands-free access to devices for the situation where the access to input device proves to be rather difficult, e.g., space exploration or provide patients who suffers ALS or other body disabilities a mean to express themselves (Trejo et al., 2006; Parini et al., 2009). BCI usually directly interacts with the cerebral activity which is evoked or self-induced and translate it into computer instructions (Wolpaw et al., 2002; McFarland and Wolpaw, 2011). There are many different approaches trying to restore the broken communication between brain and environment from many aspects. These methods include “Functional Magnetic Resonance Imaging” (fMRI) which is blood oxygenation level dependent and the invasive Electrocorticography (ECoG) as well as Electroencephalography (EEG). Comparing to other non-invasive methods, EEG provides temporal resolution up to milliseconds while it is cheaper, non-radioactive and provides high portability which makes EEG become a favorable choice when building a BCI system (Sitaram et al., 2007; Cincotti et al., 2008; Athanasiou and Bamidis, 2010; Becedas, 2012; Pistohl et al., 2012; Baranauskas, 2014).

“Steady-State Visually Evoked Potential” (SSVEP) is a kind of EEG signal generated at the visual cortex. It usually evoked by visual stimuli which flickers at the range of around 5–20 Hz while LEDs or monitors are often used as the stimuli (Herrmann, 2001; Wang et al., 2006; Friman et al., 2007; Guger et al., 2012; Zhang et al., 2012). Due to its nature of high temporal resolution of milliseconds, SSVEP possesses great potentials in real-time application while methods like PET, fMRI, and NIRS which utilize the metabolism provide good spatial resolution but poor temporal resolution; making them a less desirable candidate for real-time brain-computer communication (Wolpaw et al., 2002; Becedas, 2012; Birbaumer et al., 2013).

SSVEP is a very efficient way for a user to impose his or her will. In most situations, user who suffers from “Amyotrophic Lateral Scleroses” (ALS) or palsy tend to still have functional vision under good control while other parts of their body is disabled (Singla, 2014). Visual evoked potential is known to associate with visual stimulations and always changes according to it. Eye's sensitivity to the stimuli is the highest at the center of the vision field. It is due to the retinal cones cell concentrate at the center (Sutter, 1992; McFarland and Wolpaw, 2011). In this way, the visual evoked potential signal carries some of the properties of the stimuli which user concentrates on, such as frequency and phase of the signal. By comparing the extracted signal with the stimuli, one could tell which stimuli the user is gazing at and then translate such information into computer instructions.

To build a practical real-time BCI system, many genius minds proposed various kinds of strategies trying to increase the accuracy as well as the spelling speed of SSVEP based BCI system. Nowadays, the spelling speed of a high performance SSVEP system could achieve an ITR (Information Transfer Rate) of around 144 bits per minute (Nakanishi et al., 2014). To build a practical SSVEP BCI system, one must improve the effect of stimulus, increasing the number of stimulus, minimizing processing time while trying hard to keep the accuracy as high as possible. Since the optimal frequency to be utilized in stimuli is 5–20 Hz. To increase the number of the stimuli, it must be coded. Therefore, many coding strategies were proposed to increase the number of stimulus as well as to increase the “Signal Noise Ratio” (SNR); such methods are: “Time Division Multiple Access” (TDMA), “Frequency Division Multiple Access” (FDMA), and “Code Division Multiple Access” (CDMA) (Wei et al., 2016). It is worth noting that a practical BCI system which uses monitor to represent the stimulus would be bound to the refreshing rate of the monitor, which limited the stimulus could be implemented on a monitor. In order to tackle this problem, *Wang* proposed a strategy which can present stimulus with frequency less than half of the refreshing rate of the monitor (Wang et al., 2010). Still, the range of idea frequency to be used as stimuli is limited to 5–20 Hz, which could code about 40 stimuli with 0.2 Hz intervals when coding the stimulus with different frequencies (Herrmann, 2001). Nakanishi et al. used a coding method of methods of mixing frequency and phase and achieved 32 stimuli (Zhang et al., 2012). Chen et al. used similar method and implemented a speller with 40 targets on a monitor (Chen et al., 2015). Wei et al. used a binary encoding method, which in theory could encode stimulus as many as needed (Wei et al., 2016). However, the binary encoding method used by Wei et al. is using up to 63 bits. Such method makes the processing too long to build a practical and portable BCI system. Kimura et al. also proposed a coding strategy adopting Frequency Shift-Keying (FSK) in digital telecommunication (Kimura et al., 2013).

Performance of the EEG signal processing algorithm would also have a great influence on the performance of a BCI system. An ideal algorithm should have high accuracy and low complexity so that it could be fit into embedded devices or FPGAs (Hwang et al., 2013). After comparing the coding methods above, it would become clear that using binary coding strategy would face the problem of rather low accuracy due to the problem of “coding repetition.” While using a binary coding strategy to coding the stimuli, the beginning or the ending of the scheme is not distinguishable. For example, if a stimulus is coded with “1101,” the output of the bit stream would be an iteration of “1101.” However, since it is rather difficult to tell the ending of each segments; It is possible for the system to misinterpret the output “1101110111011101” into 1 1011 1011 1011 101 rather than 1101 1101 1101 1101. Meanwhile, the complex coding strategy used by these methods often requires a lot of computational power and thus limited their application in portable BCI devices which based on embedded device and batteries.

To mark the ending of each code, another third bit was introduced additional from the “Binary Frequency-Shifted Keying” (BFSK) used by *Kimura* which is named “Bit 2.” An experiment was conducted to evaluate its feasibility. By introducing this third bit, one could easily tell the ending of each code and thus increase the accuracy of binary encoded stimuli based BCI system.

## Materials and methods

### Modulation of FSK-SSVEP stimuli signal

**Figure 1:**
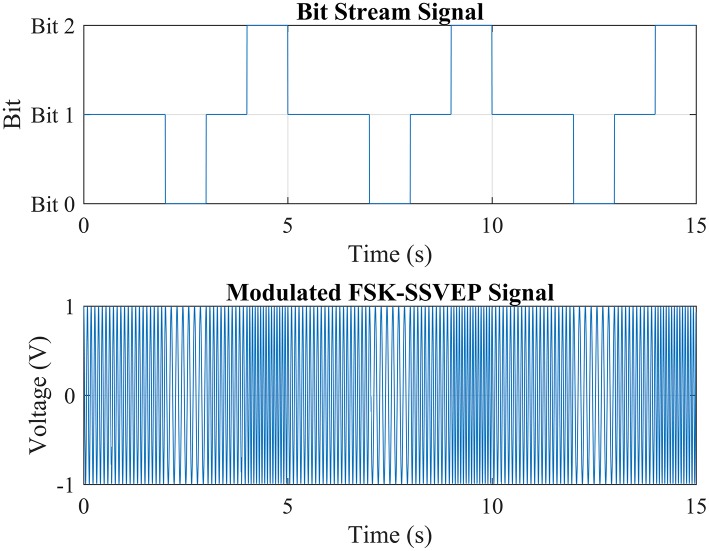
Modulation of TFSK-SSVEP signal.

Frequency-Shift Keying is a modulation method commonly used in digital communication. It shifts the frequency of a continuous binary signal to two discrete frequencies as a “space frequency” and a “mark frequency” representing “0” and “1” in binary respectively. Such modulation is called “Binary Frequency-Shift Keying” (BFSK) or FSK for short. Unlike to the traditional modulation of BFSK which is commonly seen in digital communication. In this paper, the third frequency is introduced in “Trinary Frequency-Shift Keying-SSVEP” (TFSK-SSVEP) as the “separate frequency” which represents “Bit 2” apart the bits representing “1” and “0.” This third bit marks the ending of each stimuli encoding composited by “1” and “0.” The Figure 1 showed an example of FSK-SSVEP signal modulation of three periods of bit stream “11012” with bit length of 1,000 ms and continued phase. “Bit 0,” “Bit 1,” and “Bit 2” are correspond to 7, 11, and 15 Hz respectively to achieve maxim performance (Herrmann, 2001). By introducing this third bit, the ending of a stimuli encoding could be marked and thus making it much easier to slice the bit stream into segments and thus solves the bit stream phrasing problem caused by the code repetition.

LED is an ideal candidate for visual stimuli because of its nature of fast response and thus making LED free from the limitation of refreshing rate of a monitor (Guger et al., 2012). To increase the performance of visual stimuli, white LED had been selected as visual stimulus which is controlled by square wave signal with continues phase rather than sinusoidal wave since square wave for better performance and it's easier to implement using embedded device (Cheng et al., 2001; Wang et al., 2010). It should be also noted that the length of the each bit also have a major impact on the result; the length of each bit should be no less than one period of its corresponded frequency otherwise it would difficult to tell if SSVEP signal is actually evoked.

**Figure 2:**
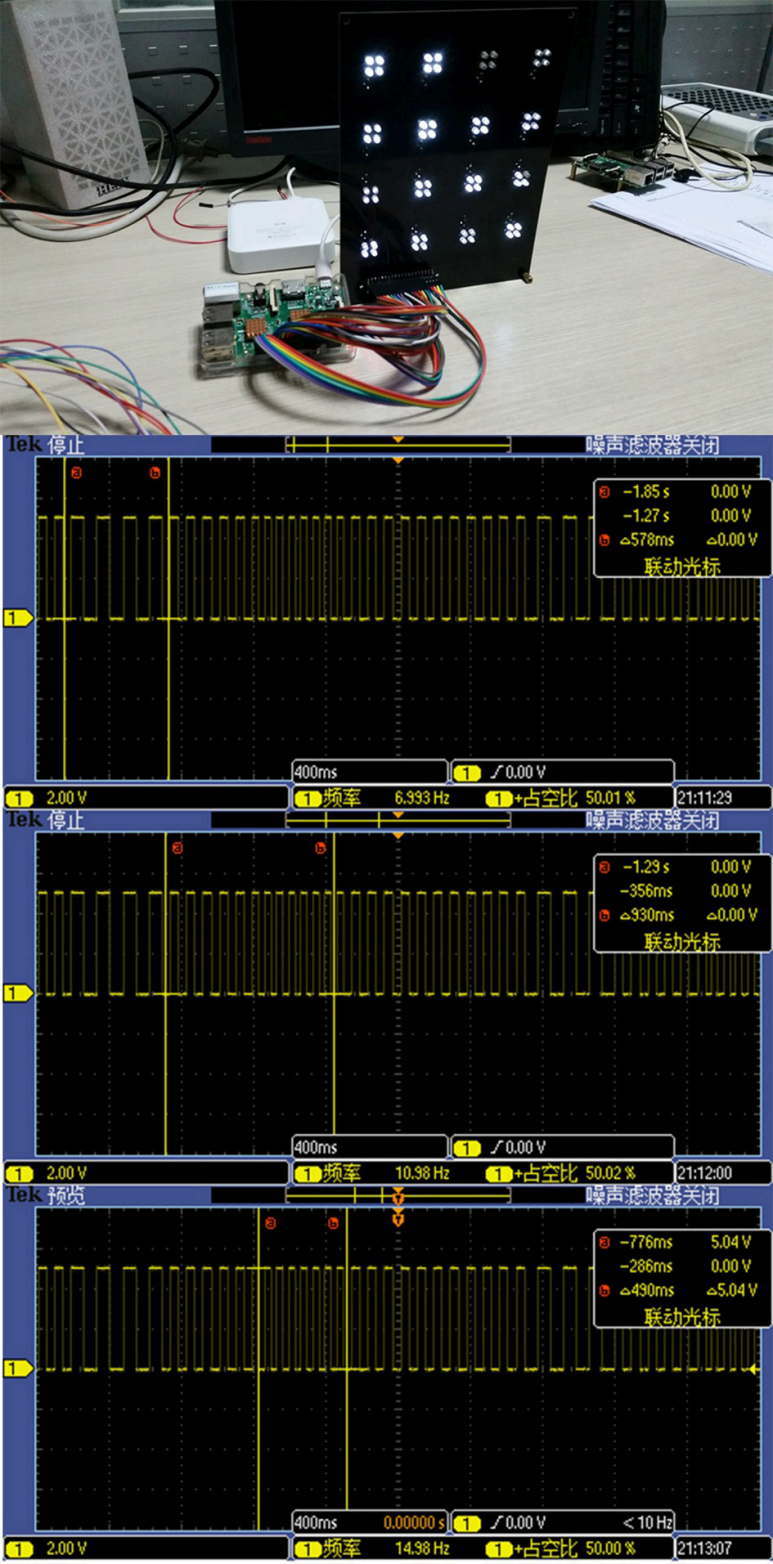
Visual stimulator powered by a Raspberry Pi Model B+ and the oscilloscope screenshots of the FSK-SSVEP control signal generated by “Raspberry Pi Model B+.” It can be inferred from the screenshots that signal generated by Pi is accurate enough to be used to control the stimulator.

During the experiment, a Raspberry Pi model B+ is used to generate the TFSK-SSVEP signal generated on its GPIO pins. Each stimulus is made of a group of four LEDs with distance of 2 cm to each other soldered to a black PCB to achieve maximum effect (Cheng et al., 2002). Each stimulus has an adjustable resistor to adjust the brightness. To suppress the interference of the halo, Figure 2 demonstrated the visual stimulator and TFSK-SSVEP signal generator using Raspberry Pi Model B+ as well as captured generated waveform.

### EEG acquisition

**Figure 3:**
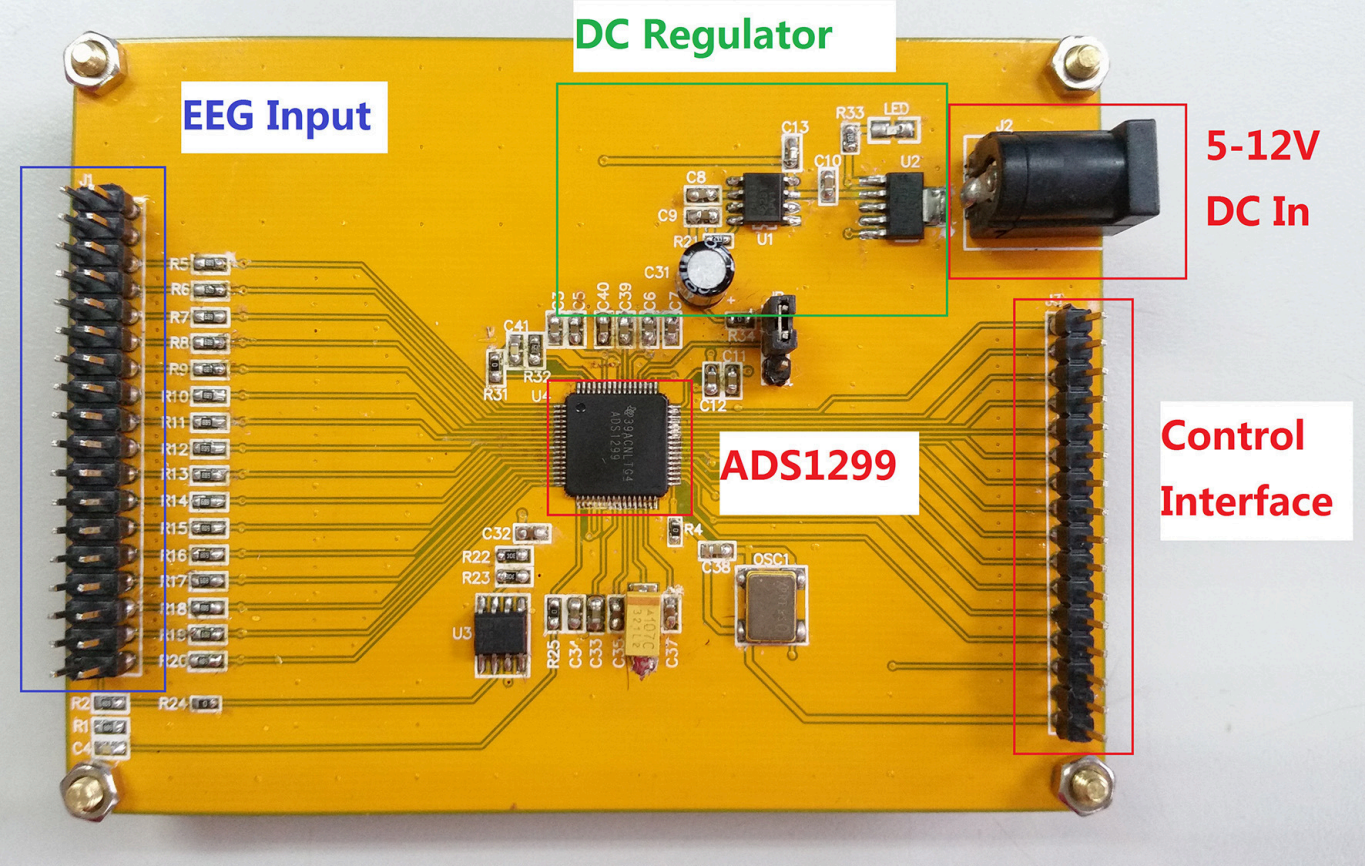
EEG acquisition board used to acquire EEG signals during the experiment.

Visual cortex is a cerebral cortex located in the occipital lobe which is in the back of the head. Therefore, the leads which are in the vicinity of the visual cortex should be selected (Sutter, 1992; Herrmann, 2001). During the experiment, signals were acquired on channel Oz, O1, O2, Pz, P3, and P4 with the sample rate of 250 SPS using device shown in Figure 3. GND is selected as reference electro node (Cheng et al., 2001; Wang et al., 2004, 2006; Parini et al., 2009; Hwang et al., 2012; Wu and Su, 2014).

### Extraction of TFSK-SSVEP signal

Since SSVEP signal carries the frequency and phase as well as other characteristics of the visual stimuli. What eventually picked up by the A/D device is the combination of strong noise and FSK-SSVEP signal. To extract the FSK-SSVEP signal, the signal acquired by A/D device must be preprocessed first before the quadrature demodulation.

**Figure 4:**
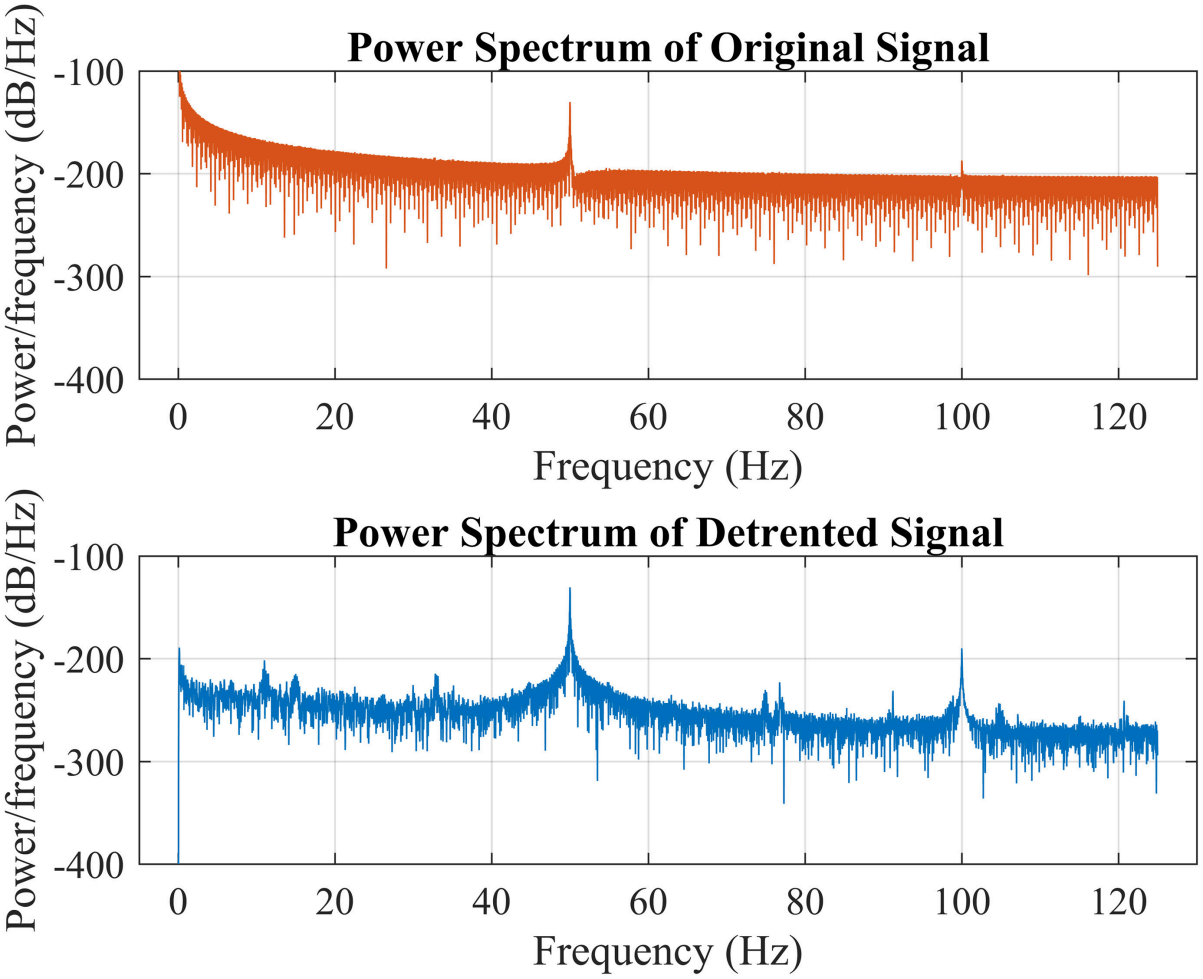
Power spectrum of the raw signal represented in red color which is unavailable to be processed without being detrended first while the blue color line showed the power spectrum of the detrended signal. Pay attention to the power spectrum around 0 Hz of both graph to get a general sensation of the characteristic of low frequency interference.

In order to do so, the raw signal is firstly detrended before band-passed using an FFT-Based FIR band-pass filter separately. The filtered signal then multiplied with their corresponded carrier frequency and then sent through a low-pass filter with a cut-off frequency at the carrier frequency. Like all bioelectrical signals, SSVEP is sensitive to disturbances such as power source interference. As shown in Figure 4 such interference usually causes trend in the signal which often will hinder the extraction of the signal and thus it must be removed and this process is called “detrending.” Conventionally, such interference could be removed by filter the signal using a high-pass filter since the interference is concentrated in low frequency domain. However, it is hard to determine the optimal parameter for the high-pass filter. Instead, the trend is removed by finding the coefficients of the polynomial which describes the trend interference statistically and subtract it from the signal.

In order to successfully demodulate the signal, the preprocessed signal should be multiplied with a sinusoidal signal which is identical to the carrier in terms of phase and frequency. For the simplicity of discussion, only the extraction process of “Bit 2” is discussed.

The modulated signal acquired using ADS1299 should contain three major components cos(*kω*_0_*n*) + cos(*kω*_1_*n*) + cos(*kω*_2_*n*). In this example, to extract ω_2_, signal is firstly filtered by bandpass filter with corresponding passband and then multiplied with the carrier signal which gives us:

(1) $$\begin{array}{l} {}[\cos\left(k\omega_{0}n\right)+\cos\left(k\omega_{1}n\right)+\cos\left(k\omega_{2}n\right)]\;\cos\left(k\omega_{2}n\right)\\ \;=\frac{1}{2}[\cos\left(k(\omega_{0}+\omega_{2})n\right)+\cos\left(k(\omega_{0}-\omega_{2})n\right)+\cos\left(k(\omega_{1}+\omega_{2})n\right)+\cos\left(k(\omega_{1}-\omega_{2})n\right)+\cos\left(k(\omega_{2}+\omega_{2})n\right)\\ \;+\;\cos\left(k(\omega_{2}-\omega_{2})n\right)]\\ \;=\;\frac{1}{2}\cos\left(k(\omega_{0}+\omega_{2})n\right)+\frac{1}{2}\cos\left(k(\omega_{1}+\omega_{2})n\right)+\frac{1}{2}\cos\left(2k\omega_{2}n\right)+\frac{1}{2}\cos\left(k(\omega_{2}-\omega_{0})n\right)\\ \;+\;\frac{1}{2}\cos\left(k(\omega_{2}-\omega_{1})n\right)+\frac{1}{2}\cos\left(0k\omega_{2}n\right) \end{array}$$

**Figure 5:**
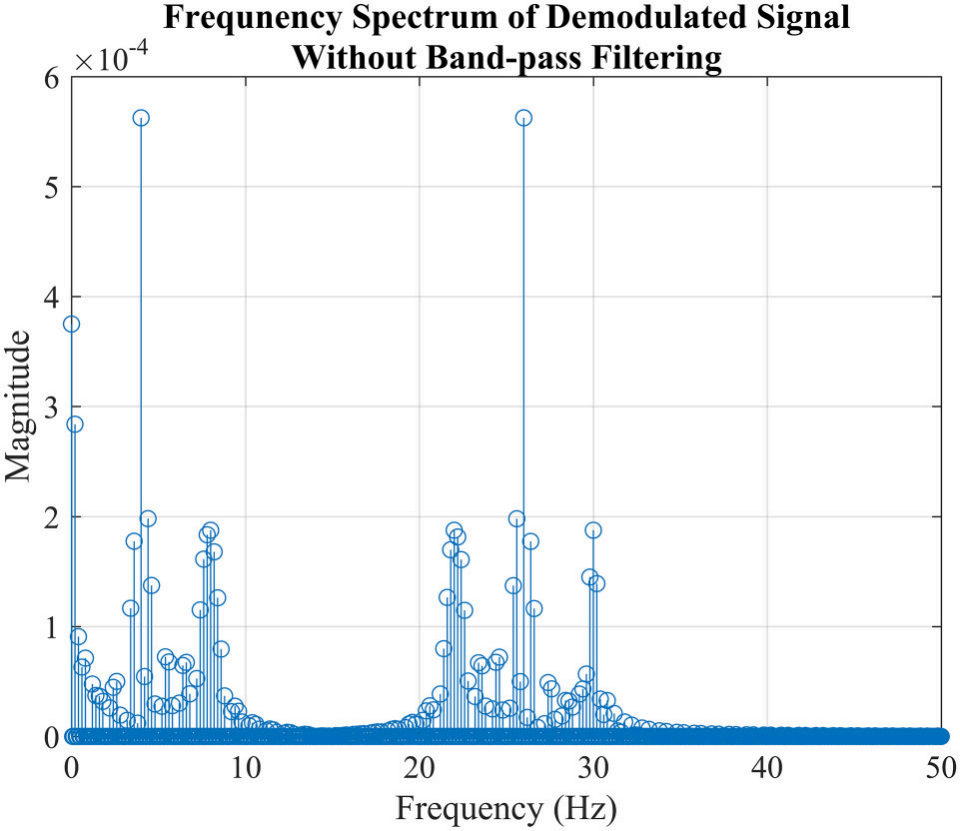
The simulation of demodulation process to extract “Bit 2” from FSK-SSVEP signal generated in the previous section “Modulation of FSK-SSVEP Stimuli Signal” which is not a real EEG signal. The figure showed the power spectrum FSK-SSVEP signal which mixed with the carrier frequency without filtered by bandpass filter. The reason why the amplitudes are not as predicted by the formula (1) is due to the composition of bit command “11012” where “Bit 1” takes a large portion.

To extract the bit stream, $\frac{1}{2}\cos\left(0k\omega_{2}n\right)$ in formula (1) needs to be recovered, Figure 5 demonstrated this idea using generated signal, From the figure, it is easy to tell that the best way to do is filtering the signal with a low pass filter with cut-off frequency around 1 Hz.

**Figure 6:**
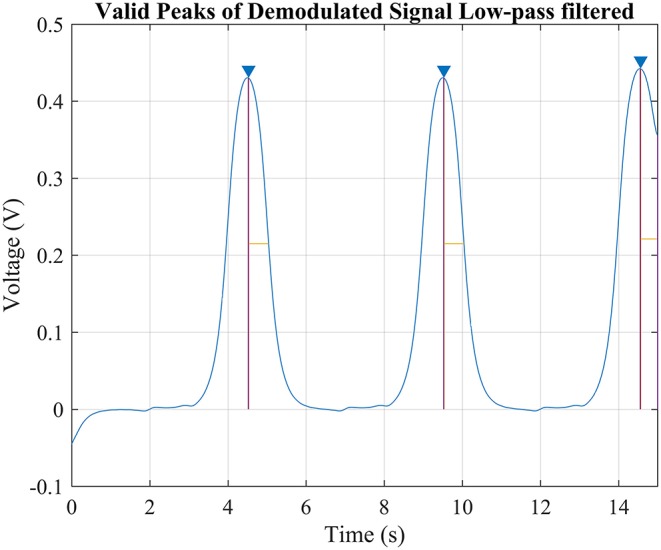
The simulation of demodulating “Bit2” signal. Since the bit length and duration of the signal is already known, there would be only 3 valid peaks possible in this signal since each command lasts 5 s and thus the highest three peaks was considered valid. Comparing the width of the peak against the known bit length would help to improve the accuracy but not necessary.

After the preprocessing, the signal contains only a relatively simple frequency component and is ready to be translated into discrete bit streams. Firstly, the valid peaks are located by calculating its derivative as well as finding the highest peaks in a time period as shown in Figure 6. The number of highest peak could be calculated when given the bit length and the duration of the signal, such kind of screening method would bypass the problem of selecting suitable threshold. The demodulated signal is then converted to bit stream by using window method. Firstly, slice the signal into segments according to the location of valid peaks of “Bit 2”; then starting from the beginning of the sliced signal and using a window which width is a bit narrower than the bit length and starting moving it toward end with the step same to bit length. If there are two valid peaks in the window, compare the amplitude of the valid peaks of “Bit 0” and “Bit 1,” discard the one which amplitude is lower and note the demodulation result as the other. In the end, append “Bit 2” to the end after the window reach the end if needed.

However, in the real application, there always would be a phase error between the EEG signal and the demodulation signal which would hinder the demodulation result and thus must be dealt with. By introducing phase error φ into the demodulation equation and calculating the trigonometric function produce the formula:

(2) $$\begin{array}{l} \cos\left(kw_{\text{x}}n\right)\cos\left(kw_{\text{c}}n-\varphi \right)=\frac{1}{2}(\text{cos}\left(2\text{k}(w_{\text{x}}+w_{\text{c}})\text{n}-\varphi \right)\\ \;+\text{cos}\varphi ) \end{array}$$

According to the formula, if there is a phase error, the demodulated signal would be amplified by cosφ and thus cause misinterpretation when interpreting the signal to bit steam. Moreover, in some extreme situations that the demodulated signal would be zero when the φ is equal to $\frac{\pi }{2}$.

To solve this problem, noticing that $\cos\left(\frac{\pi }{2}-\text{k}\omega \pi \right)=\text{sin}\;\left(\text{k}\omega \pi \right)$ which is to say that if the preprocessed signal is mixed with sin (kωπ) and cos (kωπ) separately in different channel, there will be always a channel that returns non-zero result. It is called “Quadrature Demodulation” in signal processing. Such step is necessary especially when the phase error between the demodulating signal and the TFSK-SSVEP signal is unknown.

### Optimization for real-time application

In this extraction situation above where the complete set of signal is given, using IIR filter could implement the filter with much lower filter order than FIR filter while the nonlinear phase distortion of an IIR filter could be compensated. However, in real-time application where signal is streaming and onboard MCU isn't powerful enough, it would be more effective using FFT-based overlap-add method to filter the signal. The method is capable of calculating the convolution of the signal with a FIR filter with high efficiency. When calculating the convolution, overlap-add method divide the signal *x*[*n*] into segments: $x\left[n\right]=\sum_{k}x_{k}\left[n-kL\right]$ where *L* is the length of segment. The convolution of the signal with FIR filter coefficients *h*[*n*] then could be written as:

(3) $$\begin{array}{l} y\left[n\right]=x{\left[n\right]}^{*}h\left[n\right]=\sum_{k}x_{k}{\left[n-kL\right]}^{*}h\left[n\right]\\ \;=\sum_{k}\left(x_{k}{\left[n-kL\right]}^{*}h\left[n\right]\right)=\sum_{k}y_{k}\left[n-kL\right] \end{array}$$

Where *y*_*k*_[*n* − *kL*] is equal to zero outside [1, *L* + *M* − 1] and it's equivalent to the N point circular convolution $x_{k}{\left[n-kL\right]}^{*}h\left[n\right]$ in region [1, *N*] where *N* ≥ *L* + *M* − 1. The circular convolution of *y*_*k*_[*n*] equals to *IFFT*(*FFT*(*x*_*k*_[*n*])*FFT*(*h*[*n*])).

In conclusion, the complexity of the FFT-based overlap–add method is *O*(*N*_*x*_log_2_*N*) while the complexity of traditional convolution is *O*(*N*_*x*_log_2_*N*_*x*_), *N*_*x*_ is the period of the signal and *N* is the signal length. In the case of filtering FSK-SSVEP signal where the period of the signal is low, it is certain that the signal length *N* would be lesser than the period *N*_*x*_ and thus making using overlap-add method to filter signal more effective.

## Result

**Figure 7:**
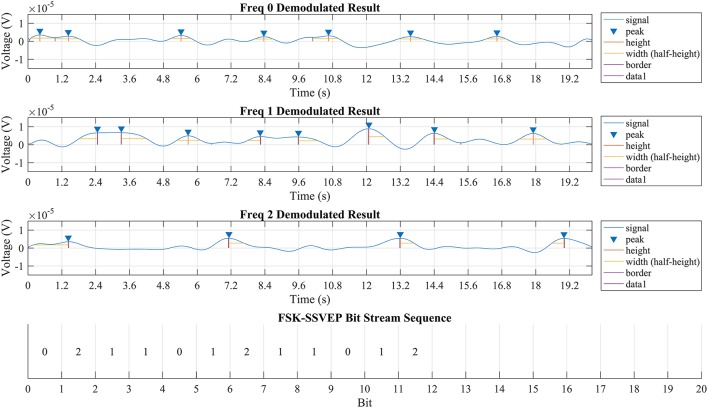
Output of quadrature demodulation using signal from Pz. The first three rows displayed the demodulation result of corresponded frequency while the valid peak is identified using a blue triangle while height and width of each peak is marked respectively. The bottom row showed part of the discrete bit stream extracted from the demodulated signals above and is identical to the bit stream “11012” with each bit length for 1,200 ms.

Figure 7 showed the demodulated signal of each frequency and the extracted bitstream respecitively. The location and amplitude of the valid peaks in demodulated signal for each frequency is also marked.

The result suggested that the brain could be modeled as a LTI system. According to the linear time-invariant theory, the output of a LTI system should be a linear map of its input except for the time delay. Such conclusion is proved to be true when compared the TFSK-SSVEP signal which stimuli transmitted against the demodulation result showed in Figure 7. “Information Transfer Rate” (ITR) of this method is still remains a high rate as conventional SSVEP (specified value depends on bit length of choosing) because this method simply uses several bits to represent a command.

## Discussion

In this paper, we had only demonstrated the feasibility of using TFSK-SSVEP as a mean to overcome the defects of stimuli in traditional SSVEP. However, there are still many improvements could be made to increase the overall performance of this method. The first could be improved is the filter, as discussed before, optimizing the filter configuration could reduce the in-band noise as much as possible and therefore improves the SNR. Currently, only a classic band-pass filter is employed to filter out the out-band noise while the in-band noise is not been processed properly (Vigario et al., 2000).

During the demodulation of certain bit, the phase of the demodulating carrier signal could be dynamically adjusted according to the phase of the FSK-SSVEP signal and compensates the phase error. Therefore, the “Quadrature Demodulation” could be simplified while increase the accuracy of a BCI system using TFSK-SSVEP. One way to achieve such objective is by calculating the phase at the designated frequency. By calculating the “Fast Fourier transform” (FFT) result of the TFSK-SSVEP signal, the signal could be transformed from time domain to frequency domain and get a sequence of components of different frequencies: $X_{k}=\overset{N-1}{\underset{n=0}{\sum }}x_{n}e^{-\frac{i2\pi \text{kn}}{\text{N}}}\;\left(k=0,1,2,\ldots ,N-1\right)$ where *x*_*n*_ is a complex number. Utilizing “Euler's formula” which gives the equation: *e*^*ix*^ = *cosx*+*isinx*, *e*^*ix*^ could be transformed to the complex panel. Therefore, for any given element in the sequence $\overset{N-1}{\underset{n=0}{\sum }}x_{n}e^{-\frac{i2\pi \text{kn}}{\text{N}}}\;\left(k=0,1,2,\ldots ,N-1\right)$ it could be transformed to the complex panel. Hence the phase at given element could be obtained by calculating the following formula: $\varphi =tan^{-1}\left(imag\left(X_{k}\right),real\left(X_{k}\right)\right)$. With the phase of the TFSK-SSVEP signal is given, the quadrature demodulation process could be replaced by a simple traditional demodulation and further more simplify this coding strategy.

**Figure 8:**
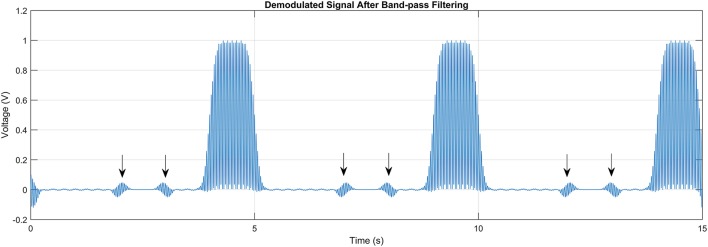
Disturbances in the signal (arrow marked) caused by the inconsistent phase during the transition between the bits in bitstream. Such disturdance would result error when extracting the bitstreams.

However, during the process of generating the TFSK-SSVEP signal, there will always introduce a very small inconsistent phase error during the transient between the bits and accumulated into a phase error which could not be ignored. As shown in Figure 8, such phase error would downgrade the performance of the demodulation process. The cause of such phase error is because the bit length could not be divided by the period of corresponding frequency of the bit stream with no remainder.

Despite this paper had proved that the visual cortex of the brain could be modeled as a LTI system, the system response function of the brain is not given. In the future work, the response of the visual cortex will be studied and more effective method to extract the bit stream will be looked into.

## Conclusion

The TFSK-SSVEP showed a new way to solve the problem of limited effective stimuli available in traditional SSVEP. It is an extremely sever problem when stimuli is presented on a monitor, limited by the refreshing rate. TFSK-SSVEP, unlike traditional SSVEP which contains only single frequency component, TFSK-SSVEP uses frequency-shift keying method to encode the visual stimuli with different and unique modulated bit stream signal. Unlike FSK in digital communication where is the bit stream signal modulated with only one carrier frequency. In FSK-SSVEP, there are 3 carrier frequency exist during the modulation of FSK-SSVEP signal. Generated FSK-SSVEP signal then used to control corresponded LED visual stimuli in square wave form to maximize the effect. The EEG signal is then sampled 250 samples per second simultaneously on Oz, O1, O2, Pz, P3, and P4 with GND electro node as the reference by ADS1299. To extract target frequency, the signal ADS1299 acquired is firstly detrended and then filtered with a band-pass filter before quadrature demodulation; eventually, demodulated signal output from the quadrature demodulation is processed to extract its valid peaks and then translated into bit streams. By using TFSK-SSVEP, problem of stimuli that had troubled SSVEP could be overcome and thus brings practice SSVEP BCI device closer into real application.

## Ethics statement

This study was carried out in accordance with the recommendations of “Guidelines of Human Experiment, Ethics Committee of Chongqing University of Post and Telecommunications” with written informed consent from all subjects. All subjects gave written informed consent in accordance with the Declaration of Helsinki and received monetary reward of 200 Chinese Yuan.

Additional Considerations:

1. “Steady-State Visually Evoked Potential” (SSVEP) is a kind of of non-invasive BCI method which does not require surgery.
2. Maximum frequency of the stimuli is 12 Hz which would not cause seizure to the subjects.
3. During the experiment, EEG acquisition device is operated on battery of 6 V.
4. Patient is protected by 4.7 k ohm patient protect resistor.

## Author contributions

XZ designed the algorithm applied in this paper and conducted the experiment. DZ provided consultants and reviewed this paper. XW helped with the experiment. XH helped in polishing the paper.

### Conflict of interest statement

The authors declare that the research was conducted in the absence of any commercial or financial relationships that could be construed as a potential conflict of interest.
